# Mesenchymal stem cells alleviate liver injury induced by chronic-binge ethanol feeding in mice via release of TSG6 and suppression of STAT3 activation

**DOI:** 10.1186/s13287-019-1547-8

**Published:** 2020-01-13

**Authors:** Yue-Meng Wan, Zhi-qiang Li, Qiong Zhou, Chang Liu, Men-Jie Wang, Hui-Xin Wu, Yun-Zhen Mu, Yue-Feng He, Yuan Zhang, Xi-Nan Wu, Yu-Hua Li, Zhi-Yuan Xu, Hua-Mei Wu, Ying Xu, Jin-Hui Yang, Xiao-Fang Wang

**Affiliations:** 1 Gastroenterology Department, The 2nd Affiliated Hospital of Kunming Medical University, Kunming City, 650101 Yunnan Province China; 2 Public Health Institute of Kunming Medical University, Kunming City, 650500 Yunnan Province China; 3 The Biomedical Engineering Research Center, Kunming Medical University, Kunming, 650500 Yunnan China; 4 Department of Pathology, The 2nd Affiliated Hospital of Kunming Medical University, Kunming City, 65010 Yunnan Province China

**Keywords:** Alcoholic hepatitis, Bone marrow mesenchymal stem cells, Oxidative stress, Neutrophil infiltration, Macrophage infiltration, Cytokines, TSG-6, STAT3 activation

## Abstract

**Background:**

Mesenchymal stem cells (MSCs) are a population of pluripotent cells that might be used for treatment of liver disease. However, the efficacy of MSCs for mice with alcoholic hepatitis (AH) and its underlying mechanism remains unclear.

**Methods:**

MSCs were isolated from the bone marrow (BM) of 4–6-week-old male C57BL/6 N mice. AH was induced in female mice by chronic-binge ethanol feeding for 10 days. The mice were given intraperitoneal injections of MSCs with or without transfection or AG490, recombinant mouse tumor necrosis factor (TNF)-α-stimulated gene/protein 6 (rmTSG-6), or saline at day 10. Blood samples and hepatic tissues were collected at day 11. Various assays such as biochemistry, histology, and flow cytometry were performed.

**Results:**

MSCs reduced AH in mice, decreasing liver/body weight ratio, liver injury, blood and hepatic lipids, malondialdehyde, interleukin (IL)-6, and TNF-ɑ, but increasing glutathione, IL-10, and TSG-6, compared to control mice. Few MSCs engrafted into the inflamed liver. Knockdown of TSG-6 in MSCs significantly attenuated their effects, and injection of rmTSG-6 achieved similar effects to MSCs. The signal transducer and activator of transcription 3 (STAT3) was activated in mice with AH, and MSCs and rmTSG-6 inhibited the STAT3 activation. Injection of MSCs plus AG490 obtained more alleviation of liver injury than MSCs alone.

**Conclusions:**

BM-MSCs injected into mice with AH do not engraft the liver, but they secrete TSG-6 to reduce liver injury and to inhibit STAT3 activation.

## Introduction

Alcoholic liver disease (ALD) is a leading cause of chronic liver disease worldwide with high morbidity and mortality [1]. According to a recent review, the prevalence of ALD in China is about 4.5%, which, quite alarmingly, is about to overtake the USA (6.2%) and European countries (6%), and has already dwarfed neighboring Japan (1.56–2.34%) [2]. ALD is a complex disease that encompasses a wide spectrum of hepatic lesions, including steatosis, alcoholic hepatitis (AH), progressive fibrosis, cirrhosis, and superimposed hepatocellular carcinoma, which may occur separately, simultaneously, or sequentially in a patient. AH is a necroinflammatory process that may cause cirrhosis in 40% of cases due to its association with the fastest progression of fibrosis, and patients without AH are at low risk of developing cirrhosis [3, 4]. Severe form of AH is associated with very high short-term mortality, about 20–50% at 3 months, and represents one of the most deadly diseases [5]. Despite cellular injury, oxidative stress, inflammation, and bacterial translocation are known driving factors for ALD, and multiple attempts have been made to improve patient outcomes, there is still no more successful treatment for this illness than alcohol abstinence and corticosteroid exposure to those with severe AH. However, these treatments are not successful in about 40% of cases [6]. Therefore, there is an urgent need to explore other new and effective therapies for this illness.

Mesenchymal stem cells (MSCs) are a population of pluripotent cells that can be obtained from many body sites, such as the bone marrow (BM) [7, 8], adipose tissue [9, 10], umbilical cord [11, 12], and placenta [13]. These cells are easy to isolate, can rapidly expand in culture and differentiate into multiple lineages of cells, and seem to be an ideal source for cytotherapy. Previous studies have reported their efficacy in treating myocardial infarction, inflammatory bowel disease, pancreatitis, burn-induced excessive inflammation, liver failure, corneal injury, and peritonitis [7–15]. The underlying mechanism for this therapy was mainly attributable to the tumor necrosis factor (TNF)-a-stimulated gene/protein 6 (TSG-6) secreted by MSCs after injection and activation [7–11, 14, 15]. However, in the literature, there is still very scarce data about the efficacy of MSCs for ALD and its underlying mechanisms up to date.

Previous studies showed that TSG-6 was able to inhibit many signaling pathways, such as the mitogen-activated protein kinase (MAPKs) pathway [11] and nuclear factor (NF)-κB pathway [15], and these signaling pathways had also been extensively studied in ALD [16, 17]. Previous studies also reported that the signal transducer and activator of transcription 3 (STAT3) signaling pathway was implicated in the pathogenesis of ALD [18], and acute ethanol intake could activate STAT3 signaling [19, 20]. However, it remains unclear how STAT3 signaling is related to AH and whether MSCs can exert their efficacy in AH through TSG-6 and/or interaction with STAT3 signaling.

Therefore, we conducted the present study to investigate the following three issues: first, to confirm the efficacy of MSCs for AH; second, to explore the association of TSG-6 with the efficacy of MSCs for AH; third, to investigate the relevance of STAT3 signaling to the treatment of AH by MSCs.

## Materials and methods

### Mice

C57BL/6 N mice were purchased from the Experimental Animal Center of Kunming Medical University. All 8–10-week-old female mice were used unless specified. All animal experiments were approved by the Ethics Committees of Kunming Medical University (No. KMMU 2019067) and were conducted according to the Guideline of Animal Care and Use Committee of Kunming Medical University.

### Isolation, characterization, and transfection of BM-MSCs

MSCs were isolated from the BM of the tibias and femurs of 4–6-week-old male C57BL/6 N mice as previously described [7, 8]. The isolation, culture, characterization, and transfection of MSCs were described in Additional file 1, Additional file 2, Additional file 3, and Additional file 4. MSCs at passages 2–3 were used in the subsequent experiments.

### Model of AH and experimental groups

AH was generated by using the Lieber-DeCarli liquid diet plus alcohol gavage according to the National Institute on Alcohol Abuse and Alcoholism (NIAAA) protocol [18], with slight modification aiming to induce more severe AH by introducing two more doses of ethanol gavage. The diets were obtained from TROPHIC Animal Feed High-Tech Co. Ltd. (Hai’an, Jiangsu, China) and were prepared fresh daily. All C57BL/6 N mice were fed a nutritionally adequate liquid control diet for 5 days (acclimatization period), then divided into the following seven groups: control, AH, AH+MSCs (AH transplanted with normal MSCs), AH+sc-MSCs (AH transplanted with MSCs transfected by Lenti-scrambled-siRNA), AH+siTSG-6-MSCs (AH transplanted with MSCs transfected by Lenti-TSG-6-siRNA), AH+recombinant mouse (rm) TSG-6 (AH followed by rmTSG-6), and AH+MSCs+AG490 (AH transplanted with normal MSCs plus AG490 injection). The AH groups were fed a liquid ethanol diet containing 5% ethanol(v/v) for 10 days, while the control group was pair-fed control diet for 10 days (modeling period).

### Experimental treatment

At days 3, 6, and 11 of the modeling period, mice in the AH groups were gavaged a single dose of ethanol (31.5% ethanol(v/v), 400 μl/mouse), while mice in the control group were gavaged isocaloric dextrin maltose (45%(w/v), 400 μl/mouse). At day 10, MSCs with or without transfection were intraperitoneally (i.p.) administered to the AH+MSCs, AH+sc-MSCs, AH+siTSG-6-MSCs, and AH+MSCs+AG490 groups (5 × 10^6^ cells/mouse); following injection of MSCs, an additional dose of AG490 (Tocris, Bristol, UK) was given to the AH+MSCs+AG490 group (20 μg/mouse, i.p.); rmTSG-6 (R&D, Minneapolis, MN, USA) was injected to the AH+rmTSG-6 group (10 μg/mouse, i.p.); equal volumes of saline were administered to the control or AH group per protocol requirement. The gavage was always performed in the morning, and mice were then maintained on control or ethanol diet. After ethanol gavage, mice were lethargic and tachypneic, but they recovered within 4–6 h. The mice were always anesthetized 9 h post the last gavage with samples of blood, peritoneal fluid lavage, and liver tissues harvested for further analyses (Additional file 1).

### Other reagents and methods

Other assays are described in Additional files 1 and 9, including blood chemistry, liver histology and immunohistochemistry, flow cytometry, myeloperoxidase (MPO) activity assay, enzyme-linked immunosorbent assay (ELISA), reverse transcription quantitative PCR (RT-qPCR) and Western-blot assays, and hepatic oxidative stress analysis.

### Statistical analysis

Data are expressed as means ± standard deviation (SD) and were analyzed using GraphPad PRISM software, version 8.02 (GraphPad Software Inc., La Jolla, CA, USA). A Student’s *t* test (for two group comparisons) or a Kruskal-Wallis one-way ANOVA followed by Tukey’s post hoc test (for three or more group comparisons) was used for statistical analyses. A value of *P* < 0.05 was considered statistically significant.

## Results

### Intraperitoneal injection of MSCs ameliorated ethanol-induced liver injury

**Fig. 1:**
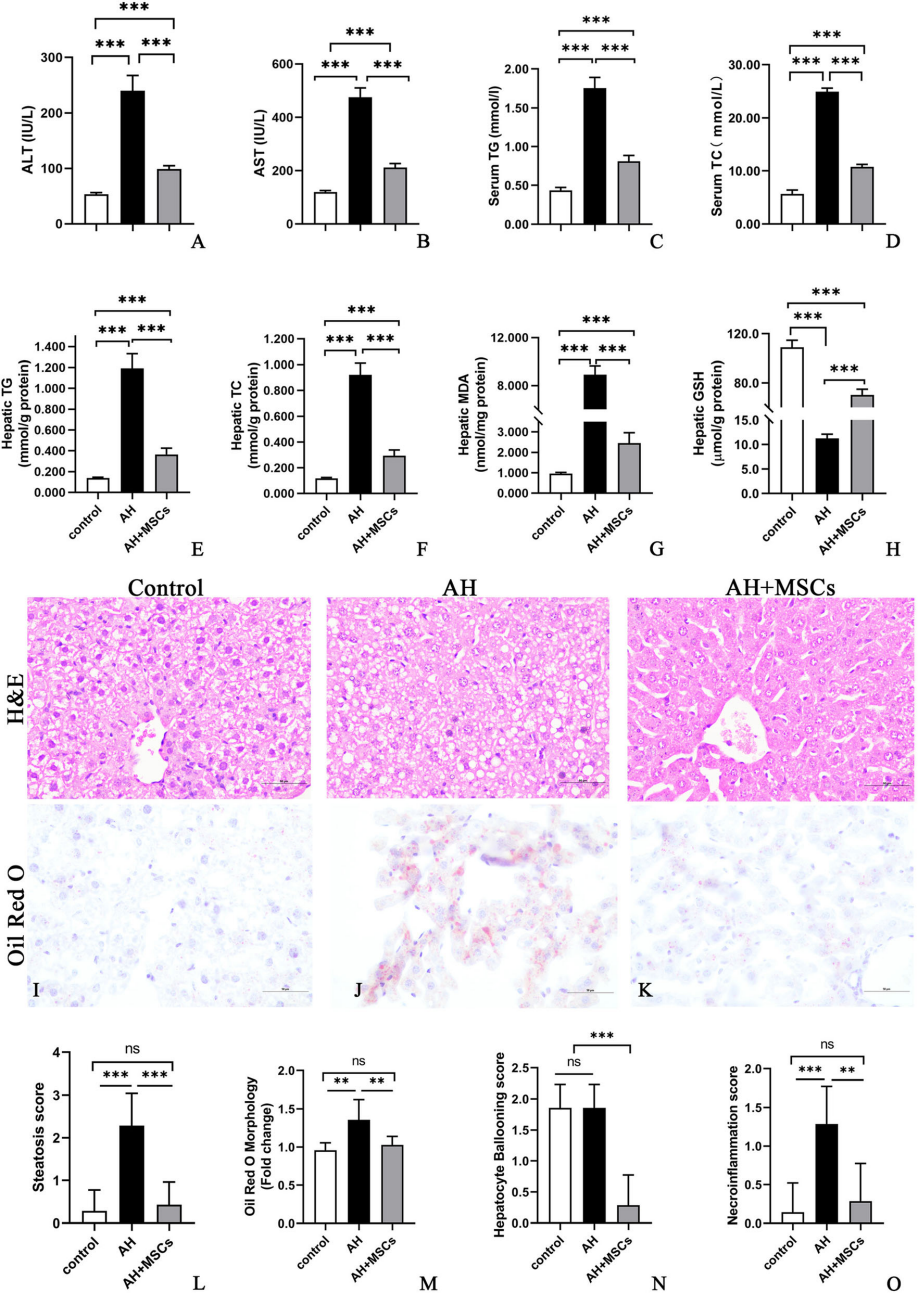
**a**–**o** MSCs ameliorated ethanol-induced liver injury (**a**, **b**), lipid dysregulation (**c**–**f**), oxidative stress (**g**, **h**), hepatic steatosis, hepatocyte ballooning, and necroinflammation (**i**–**o**). Bars 50 μm, seven mice per group were used. *P < 0.05, **P < 0.01, ***P < 0.001

**Fig. 2:**
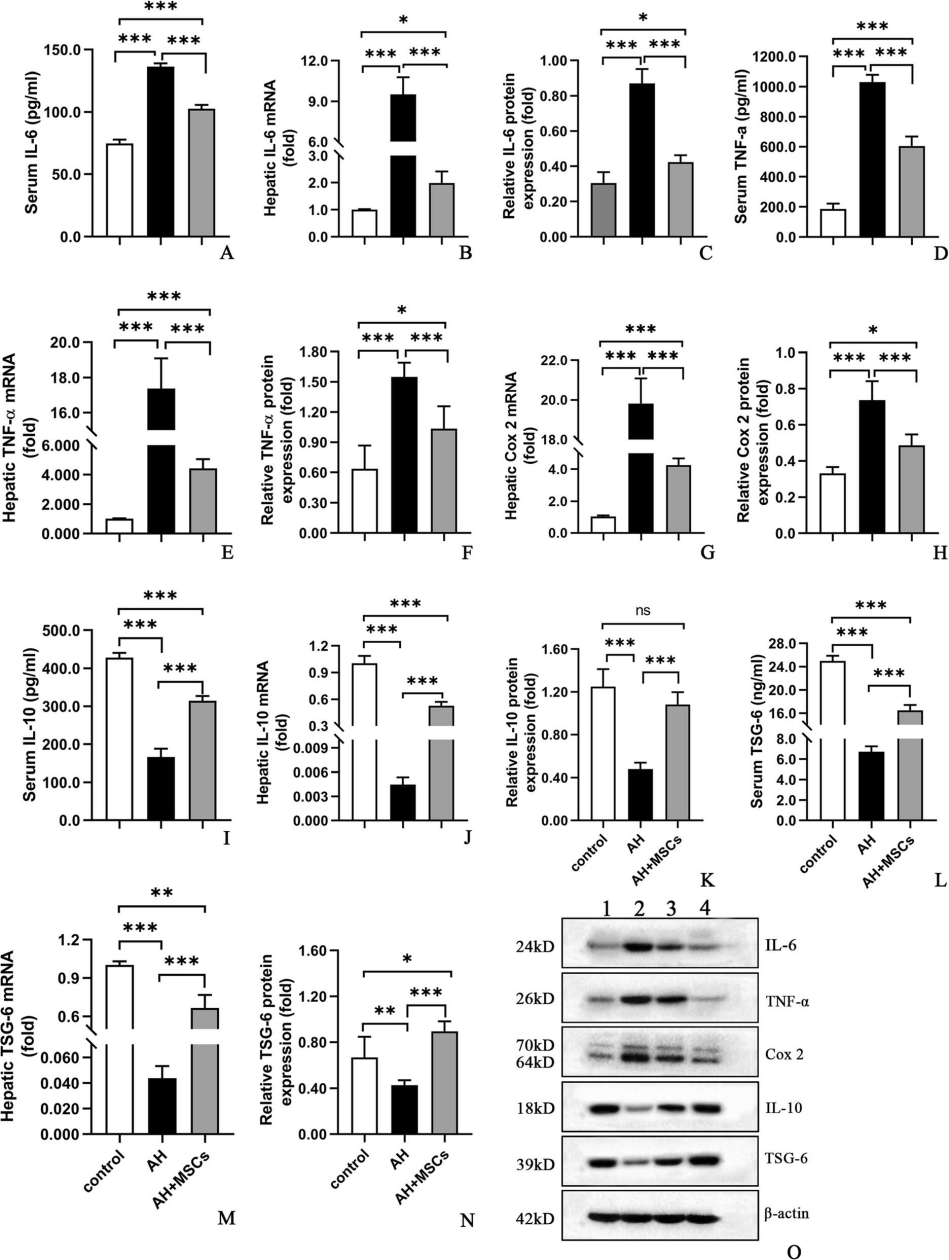
**a**–**o** MSCs decreased IL-6 (**a**–**c**), TNF-α (**d**–**f**), and Cox 2 (**g**, **h**), but increased IL-10 (**i**–**k**) and TSG-6 (**l**–**n**) expressions in the blood and liver; Western blot analysis (**o**): lane 1, control; lane 2, AH; lane 3, AH+MSCs; lane 4, AH+MSCs+AG490. Five mice per group were used. *P < 0.05, **P < 0.01, ***P < 0.001

Until day 11, similar body weight gains were observed among all groups, though liver/body weight ratio was higher in the AH group compared to the control group (Additional file 2: Figure S1j, k). i.p. injection of MSCs (5 × 10^6^cells/mouse) significantly improved the liver injury parameters such as liver/body weight ratio (Additional file 2: Figure S1k,l), serum alanine aminotransferase (ALT) (Fig. 1a), aspartate aminotransferase (AST) (Fig. 1b), total triglyceride (TG), total cholesterol (TC) levels (Fig. 1c, d), hepatic TG and TC concentrations (Fig. 1e, f), hepatic malondialdehyde (MDA) and glutathione (GSH) (Fig. 1g, h), hepatic steatosis, hepatocyte ballooning, necroinflammation, and corresponding histology scores (Fig. 1i–o) as well as hepatic infiltration by neutrophils (Additional file 5: Figure S4) and monocytes/macrophages (Additional file 6: Figure S6). No significant signs of liver injury except hepatocyte ballooning were detected in the control group (Fig. 1i, n). Consistent with these data, we observed that i.p. injection of MSCs (5 × 10^6^cells/mouse) markedly dampened the systemic and hepatic inflammatory responses as reflected by reduced proinflammatory cytokines (i.e., interleukin (IL)-6, TNF-α, cyclo-oxygenase (Cox)-2) (Fig. 2a–h) and elevated anti-inflammatory cytokines (i.e., IL-10, TSG-6) (Fig. 2i–n) in the AH+MSC group as compared to the AH group.

### Low frequency of MSCs engrafted into the inflamed liver

To evaluate the migratory ability of i.p. injected MSCs, we analyzed the hepatic expression of SRY protein by immunofluorescent assay, since SRY was a male sex determinant gene and its expression in female tissue indicated the presence of allogenic cells. Twenty-four hours after transplantation of MSCs derived from male mice, SRY protein could be sparsely seen in the liver samples of female mice in the AH+MSCs group (Additional file 7: Figure S6a). In control mice, no SRY protein expression could be detected. Moreover, SRY protein expression could be hardly detected in the liver of mice in the AH+MSCs-sc or AH+siTSG-6-MSCs groups that were transplanted with MSCs transfected by lentivirus (Additional file 7: Figure S6b, c). All these data demonstrated that low frequency of MSCs engrafted into the inflamed liver and the therapeutic efficacy of MSCs was unlikely due to the engraftment of MSCs into the liver, but possibly due to certain paracrine factors.

### Intraperitoneal injection of MSCs attenuated ethanol-induced liver injury through TSG-6

**Fig. 3:**
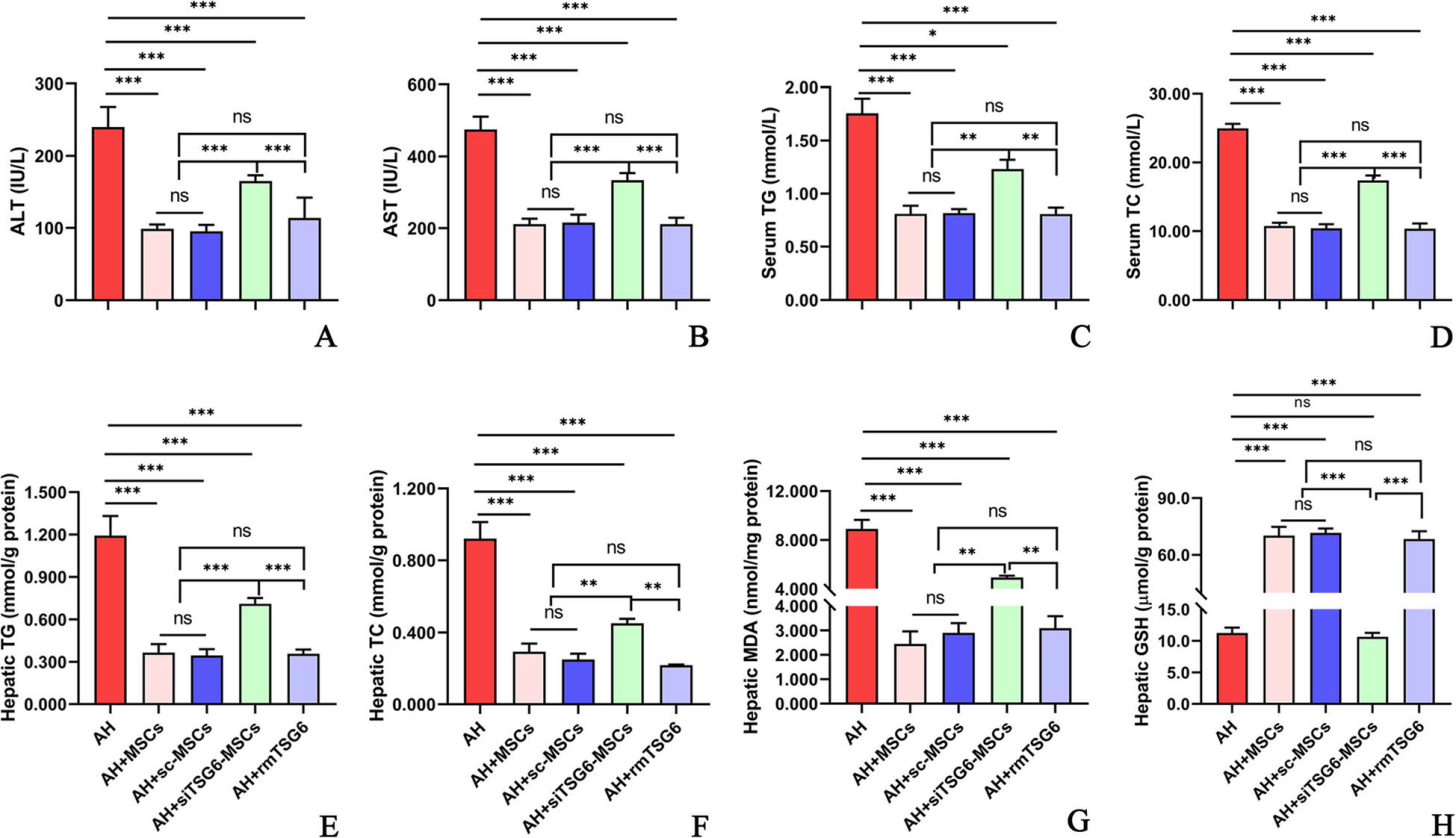
**a**–**h** TSG-6 knockdown in MSCs attenuated their hepatoprotective effects, and exogenous administration of rmTSG-6 mimicked their effects in AH. Liver injury (ALT, AST; **a**, **b**). Serum and hepatic TG and TC (**c**–**f**). Hepatic oxidative stress (MDA, GSH; **g**, **h**). Seven mice per group were used. *P < 0.05, **P < 0.01, ***P < 0.001

**Fig. 4:**
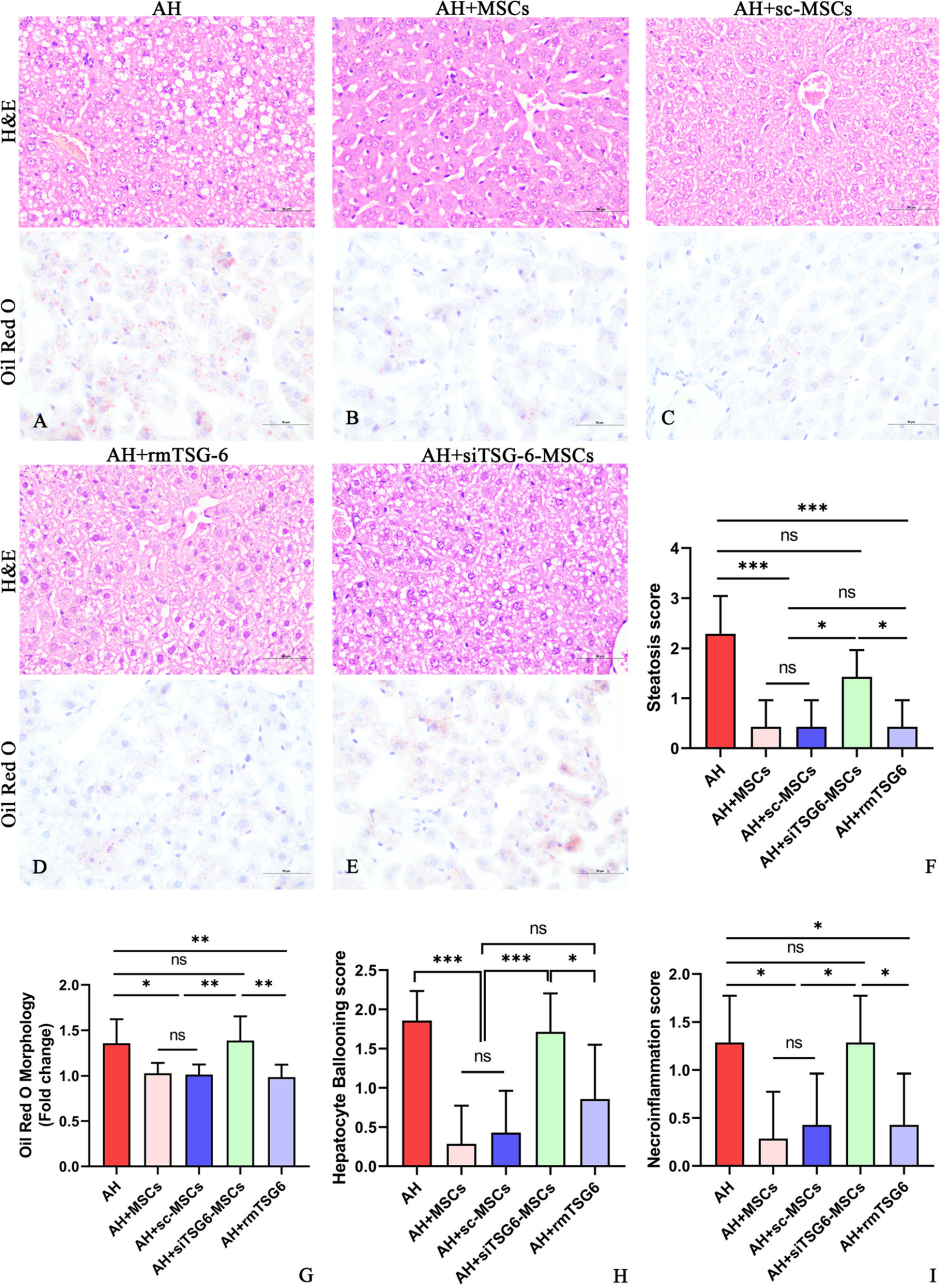
**a**–**i** Hepatic steatosis, hepatocyte ballooning, necroinflammation, and corresponding histology scores after H&E and Oil Red O staining (**f**–**i**). Bars 50 μm. Seven mice (**f**-**i**) per group were used. *P < 0.05, **P < 0.01, ***P < 0.001

**Fig. 5:**
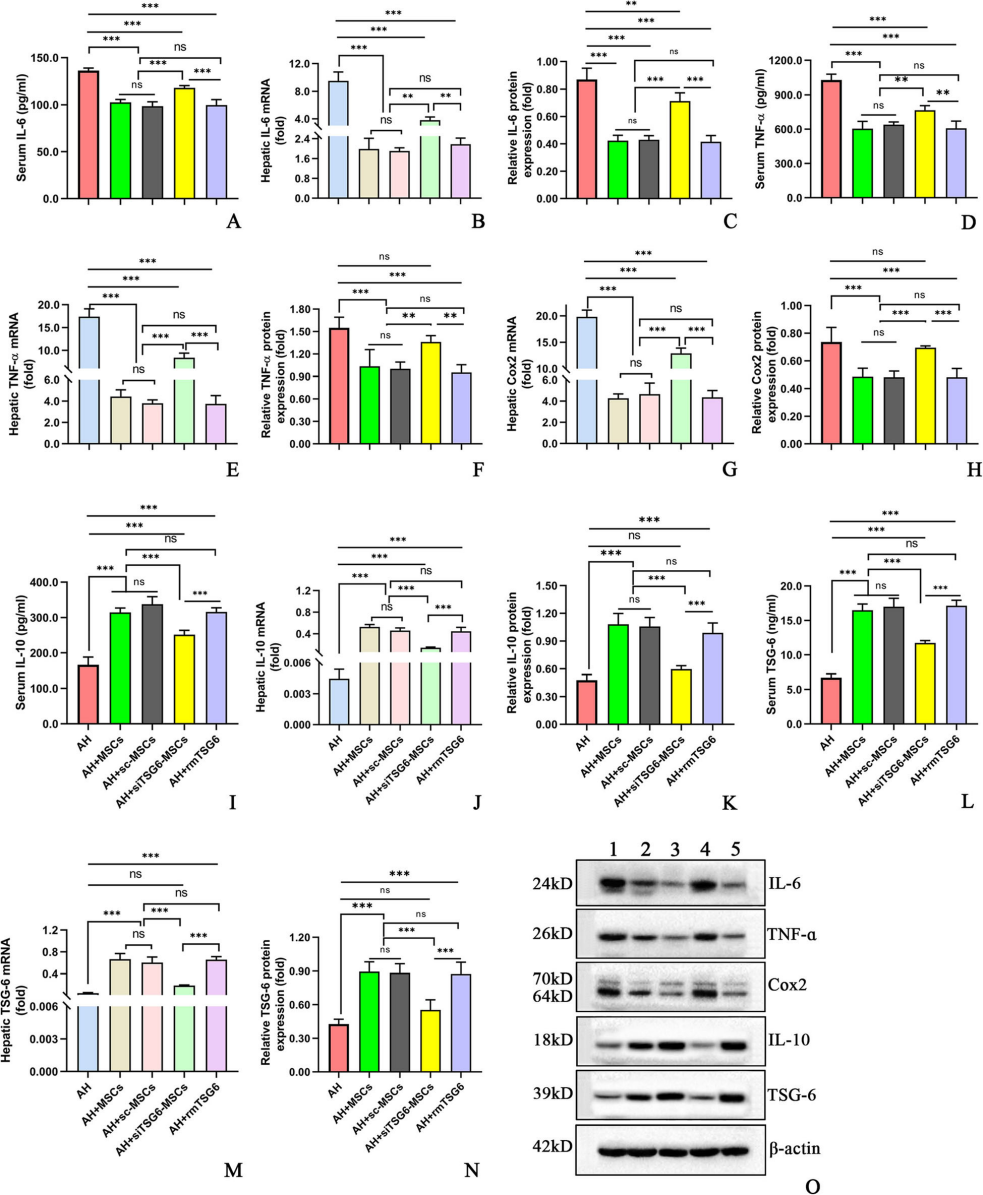
**a**–**o** TSG-6 knockdown in MSCs weakened their immunomodulatory effects, and exogenous administration of rmTSG-6 replicated their effects in AH. Serum and hepatic IL-6 (**a**–**c**), TNF-ɑ (**d**–**f**), Cox2 (**g**, **h**), IL-10 (**i**–**k**), and TSG-6 (**l**–**n**) mRNA and protein; Western blot results of hepatic tissue (**o**): lane 1, AH; lane 2, AH+MSCs; lane 3, AH+sc-MSCs; lane 4, AH+siTSG-6-MSCs; lane 5, AH+rmTSG-6. Five mice per group were used. *P < 0.05, **P < 0.01, ***P < 0.001

We next examined the mechanism by which MSCs exerted their therapeutic effects in AH. Since numerous studies reported that MSCs acted through secreting TSG-6 in other disease models [7–11, 14, 15], we attempted to investigate whether MSCs also worked via secreting TSG-6 by the strategy of TSG-6 knockdown and mimic. Compared to the untreated AH mice, i.p. injection of 5 × 10^6^ cells/mouse caused a significant reduction of liver/body weight ratio (Additional file 2: Figure S1k,l), liver enzymes (ALT, AST; Fig. 3a, b), and blood and hepatic lipids (TG, TC; Fig. 3c–f). However, these effects were markedly weakened after i.p. injection of 5 × 10^6^ siTSG-6-MSCs/mouse, but were not affected after i.p. injection of 5 × 10^6^ sc-MSCs/mouse. Meanwhile, i.p. administration of rmTSG-6 (10 μg/mouse) showed comparable effects to that of MSCs or sc-MSCs injection (Fig. 3a–f; Additional file 2: Figure S1 l). Oxidative stress is a known driving factor for ALD. We observed that i.p. injection of 5 × 10^6^ MSCs/mouse markedly lowered the hepatic MDA but increased the hepatic reserve of GSH in comparison to the untreated mice, which was significantly negated by injection of siTSG-6-MSCs but not by sc-MSCs, and administration of rmTSG-6 mimicked the effects of MSCs or sc-MSCs (Fig. 3g, h). Similarly, i.p. injection of 5 × 10^6^ MSCs/mouse prominently alleviated the hepatic steatosis, hepatocyte ballooning, necroinflammation and corresponding histology scores (Fig. 4a–i), and infiltration by neutrophils (Additional file 5: Figure S4) and monocytes/macrophages (Additional file 6: Figure S5), which were obviously abolished by injection of siTSG-6-MSCs but not sc-MSCs, and infusion of rmTSG-6 replicated the effects of MSCs or sc-MSCs. In line with these findings, siTSG-6-MSCs but not sc-MSCs markedly decreased the IL-6, TNF-α, and Cox 2 levels (Fig. 5a–h) and enhanced the IL-10 and TSG-6 (Fig. 5i–n) levels in the mouse blood and inflamed liver. And exogenous administration of rmTSG-6 produced similar results to those of MSCs and sc-MSCs, but not to siTSG6-MSCs (Fig. 5a–n).

### MSCs reduced ethanol-induced liver injury through secreting TSG-6 to inhibit STAT3 signaling

**Fig. 6:**
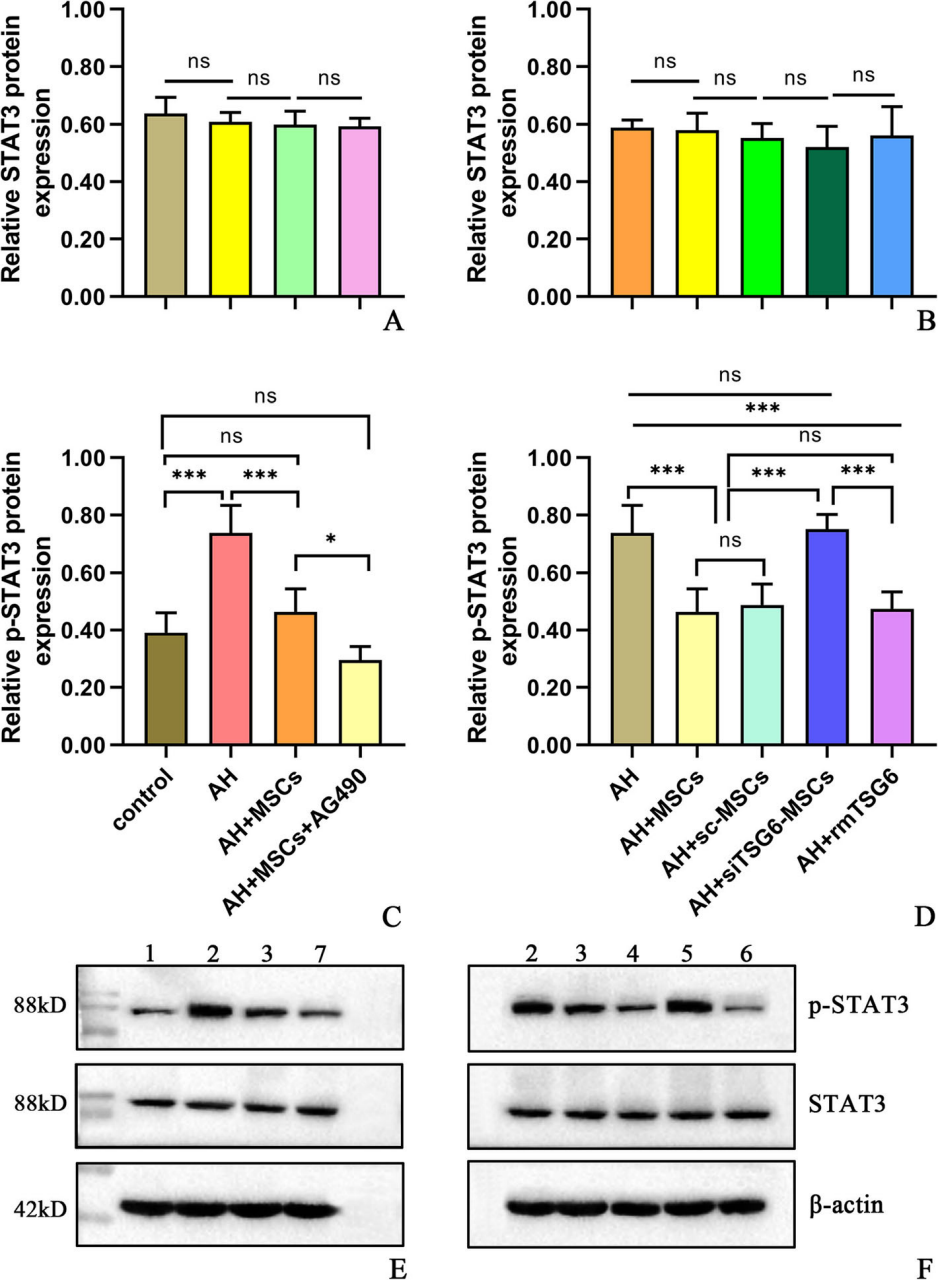
**a**–**f** Hepatic STAT3 (**a**, **b**), p-STAT3 levels (**c**, **d**), and Western blot results (**e**, **f**): lane 1, control; lane 2, AH; lane 3, AH+MSCs; lane 4, AH+sc-MSCs; lane 5, AH+siTSG-6-MSCs; lane 6, AH+rmTSG-6; lane 7, AH+MSCs+AG490. Five mice per group were used. *P < 0.05, **P < 0.01, ***P < 0.001

To investigate the potential mechanism underlying the effects of MSCs on AH in mice, we evaluated the STAT3 signaling, since STAT3 was reported to play a significant role in ALD [18–20]. We observed that STAT3 signaling was significantly activated in the AH group compared to the normal control group, as reflected by increased phosphorylated STAT3 (p-STAT3) level, though the STAT3 level was not altered (Fig. 6a, c, e). After i.p. administration of MSCs or sc-MSCs but not siTSG-6-MSCs (5 × 10^6^ cells/mouse), p-STATs level was significantly reduced, but STAT3 level was unchanged in comparison to the untreated mice (Fig. 6b, d, f). Moreover, i.p. injection of rmTSG-6 produced similar suppression of p-STAT3 to that of MSCs or sc-MSCs, while STAT3 was not affected (Fig. 6b, d, f). Interestingly, i.p. infusion of AG490 (a STAT3 signaling inhibitor) plus MSCs further suppressed p-STAT3 but not STAT3 level in comparison to MSC administration alone (Fig. 6a, c, e), which also resulted in much more pronounced improvement of AH (Additional file 8: Figure S7). All these data supported that MSCs acted through TSG-6 that could in turn inhibit the STAT3 signaling.

## Discussion

In the present study, five important findings were presented here. First, we successfully modified the NIAAA model (i.e., chronic-binge model) and induced more severe form of ALD by introducing three ethanol gavages at days 3, 6, and 11 of the modeling period. And the peak ALT and AST levels in our study were 279 IU/L and 515 IU/L, respectively, as opposed to 250 IU/L ALT and 420 IU/L AST in the NIAAA model [18]. Second, we demonstrated that i.p. transplantation of MSCs was effective in treating AH in mice. Third, we provided evidence that MSCs exerted their efficacy in treating AH through secretion of TSG-6. Fourth, we showed that STAT3 signaling was activated in AH, and MSCs could inhibit STAT3 signaling through TSG-6. Fifth, our results suggested that further inhibition of STAT3 signaling by AG490 could enhance the efficacy of MSCs in AH.

Currently, the NIAAA model for ALD has been widely used [18, 21], so we adopted this model to study the efficacy of MSCs. In this study, we observed that i.p. transplantation of MSCs alleviated ethanol-induced AH, and the ALT and AST levels were significantly reduced in mice receiving MSC transplantation compared to the untreated ones (Fig. 1a, b), showing a hepatoprotective effect. Previous studies reported that the blood and hepatic TG and TC levels were elevated in AH mice [18, 22]. Consistent with these studies, the serum and hepatic TG and TC were significantly higher in the AH group than in the control group, which were markedly reduced after injection of MSCs (Fig. 1c–f). In addition, the H&E and Oil Red O staining also illustrated obvious improvement of hepatic steatosis after MSC therapy (Fig. 1i–m), exhibiting an anti-steatogenic effect. Oxidative stress plays an important role in the pathogenesis of ALD, and many studies reported that oxidative stress and production of reactive oxygen species could induce steatosis, inflammatory cell infiltration, hepatomegaly, fibrosis, and cirrhosis [18, 23, 24]. In agreement with these studies, we noted that MDA, a product of oxidative stress and lipid peroxidation, was much higher, whereas GSH, an antioxidant, was much lower in the AH group than in the control group (Fig. 1g, h). However, MSC therapy decreased the MDA level but increased the GSH level (Fig. 1g, h), displaying an antioxidative effect.

Previous studies showed that recruitment and activation of neutrophils and macrophages played a significant role in the development of ethanol-induced liver injury [18, 22]. In concert with these studies, the hepatic numbers of neutrophils and monocytes/macrophages were much greater in the AH group than in the control group, which were prominently reduced after MSC therapy (Additional files 5 and 6: Figure S4, 5), illustrating an anti-inflammatory effect. An increase of proinflammatory cytokines like TNF-ɑ, IL-1, and IL-6 in the blood and hepatic tissues is a key feature of ALD [18, 22, 25]. In conformity to these studies [18, 22, 25], the TNF-ɑ and IL-6 levels were significantly higher in the AH group than in the control group (Fig. 1a–f). As for anti-inflammatory cytokines, our study showed that the serum and hepatic IL-10 levels were decreased in the AH group when compared to the control group (Fig. 1i–k), which was at odds with a previous study that reported an increase rather than decrease of serum IL-10 and hepatic IL-10 mRNA in AH mice [22]. Nonetheless, the serum IL-10 level in the previous study was less than 50 pg/ml in contrast to about 300 pg/ml in our study, which may be explained by the different animal modeling process between our study and the previous one (three ethanol gavage vs. one ethanol gavage) that may lead to different severity or stage of AH. Moreover, our study also demonstrated a similar trend of TSG-6 to IL-10 in both the serum and hepatic levels (Fig. 1l–n). After therapy with MSCs, the proinflammatory cytokines (i.e., TNF-α, IL-6) were diminished, whereas the anti-inflammatory cytokines (IL-10, TSG-6) were enhanced, showcasing an immunomodulatory effect.

The above findings provided strong evidences that BM-MSCs were effective in treating AH. We next sought to explore the underlying mechanism from pro and con two aspects by TSG-6 knockdown and exogenous injection. After knockdown of TSG-6 using TSG-6 siRNA, the efficacy of MSCs was significantly decreased; in contrast, knockdown of TSG-6 using scrambled siRNA as a strict control did not affect the efficacy of MSCs; and exogenous administration of rmTSG-6 achieved comparable effects to MSC therapy (Figs. 3, 4, and 5). These comprehensive data proved robustly that MSCs exerted their efficacy in alleviating AH in mice via secreting TSG-6, which was in line with the previous studies [7–11, 14, 15].

Previous studies demonstrated that STAT3 signaling played a very important role in liver injury, hepatic inflammation, steatosis, regeneration, and neutrophil trafficking [26–28], and acute ethanol intake could activate STAT3 signaling in monocytes [19, 20]. Therefore, we hypothesized that hepatic STAT3 signaling was closely related to liver injury, steatosis and inflammation, and hepatic neutrophil and monocyte/macrophage infiltration in AH, and hepatic STAT3 signaling might be a therapeutic target of MSC treatment. However, to our best knowledge, there is no study investigating the effects of MSCs or TSG-6 on hepatic STAT3 signaling in ALD. Consistent with previous studies [19, 20], our study found that hepatic STAT3 signaling was prominently activated in AH, as the p-STAT3 level was much higher in the AH mice than in the control mice (Fig. 6c, e). And MSC therapy significantly inhibited the hepatic STAT3 signaling, as the p-STAT3 level in mice receiving MSC therapy was markedly decreased compared to the untreated mice, although the STAT3 level was not changed (Fig. 6a, c, e). Using the strategy of TSG-6 knockdown in MSCs and exogenous administration of TSG-6, our data suggested that the inhibition of MSCs on hepatic STAT3 signaling was mainly mediated by TSG-6, and TSG-6 could directly suppress STAT3 signaling (Fig. 6b, d, f). Many cytokines and growth factors can activate STAT3 signaling, including IL-6, IL-10, and IL-22. However, STAT3 activation by different cytokines may elicit opposing effects. For example, STAT3 activation by IL-6 potentiates proinflammatory responses in peritoneal macrophages [29], whereas STAT3 activation by IL-10 dampens lipopolysaccharide (LPS)-induced inflammatory responses in Kupffer cells [30, 31], and STAT3 activation by IL-22 ameliorates ethanol-induced liver injury [18]. STAT3 activation may also have opposing functions in the liver. While hepatocyte-specific STAT3 knockout alleviates liver inflammation in acute liver injury induced by carbon tetrachloride [32] or ethanol ingestion [33], it enhances liver inflammation in Con A-induced T cell hepatitis [34] or in LPS-induced liver injury [35], suggesting that hepatic STAT3 activation may serve as either a proinflammatory or an anti-inflammatory signal according to the models. The findings in our study indicated that MSCs- or TSG6-induced suppression of hepatic STAT3 activation might play a beneficial role in relieving liver injury, liver inflammation, and hepatic steatosis and in modulating the inflammatory responses (Figs. 3, 4, and 5). Furthermore, we observed enhanced efficacy of MSC therapy in alleviating AH after adding AG490, an inhibitor for STAT3 signaling, to MSC therapy (Additional file 8: Figure S7a-h). This finding provided direct evidence that suppression of hepatic STAT3 signaling by MSCs through TSG-6 may be the underlying mechanism of its efficacy for AH in mice (Fig. 6).

There are several limitations to the present study. First, we did not provide sufficient evidence supporting the direct linkage between suppression of hepatic STAT3 activation by MSCs through TSG-6 and improvement of liver injury, hepatic steatosis, oxidative stress, neutrophil and monocyte/macrophage infiltration, and change of serum and hepatic cytokines. Second, we did not identify the cellular source responsible for STAT3 activation, as it may be liver parenchymal cells, hepatic neutrophils, or macrophages. However, our study is the first one that investigates the efficacy of MSCs on AH in mice, and we have five important findings as presented before that may be helpful in developing new therapies for more severe AH.

## Conclusion

**Fig. 7:**
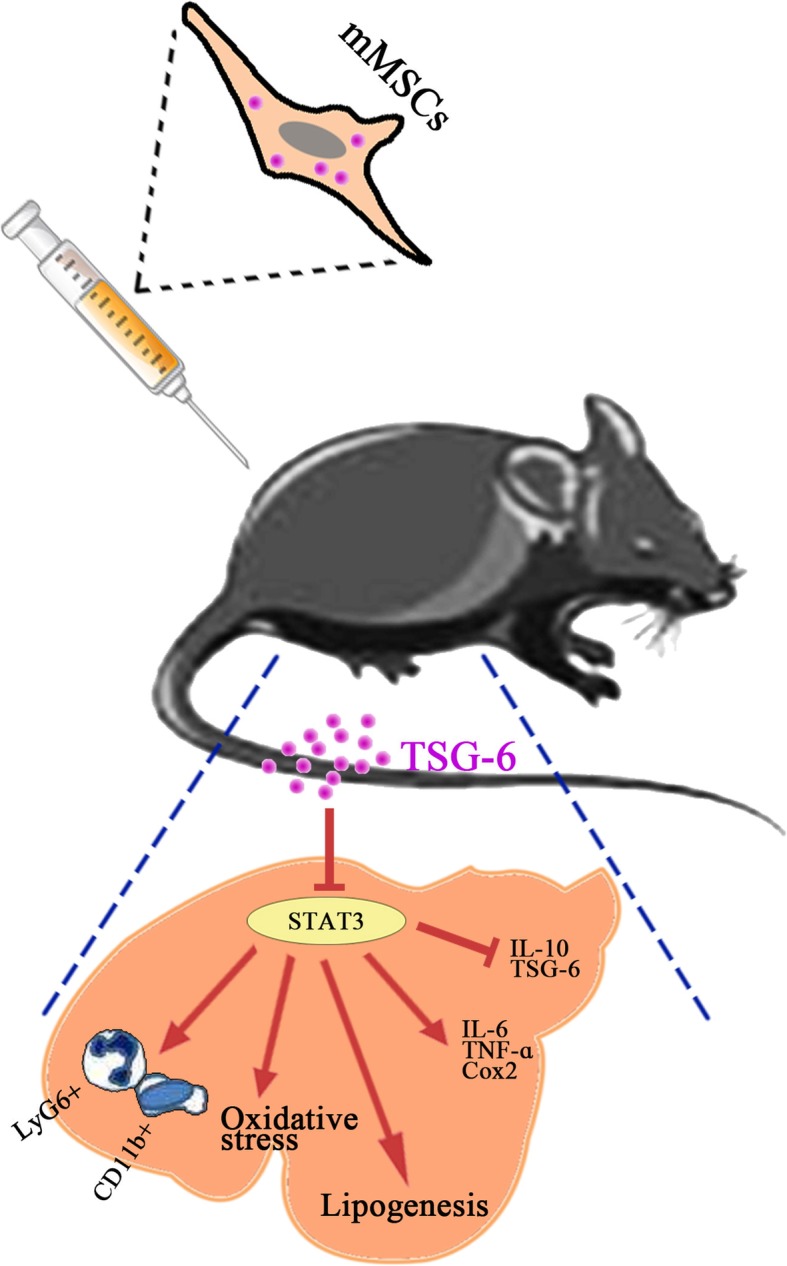
Schematic overview of mouse MSC activities in the treatment of alcoholic hepatitis

In conclusion, our study clearly demonstrated that BM-MS were effective in treating AH, and they exhibited hepatoprotective, anti-steatogenic, antioxidative, anti-inflammatory, and immunomodulatory effects through secreting TSG-6 and inhibiting hepatic STAT3 signaling. Based on previous studies [19, 20, 26–28] and our findings, we propose that MSCs secreting TSG-6 to suppress hepatic STAT3 signaling may be one underlying mechanism of the efficacy of MSCs for AH in mice (Fig. 7). These findings may help in the development of stem cell therapies for the treatment of ALD.

## Supplementary information

**Additional file 1:.** Supplementary materials. Mouse bone marrow-derived mesenchymal stem cells: isolation, culture, characterization and transfection; sample collection, storage and various assay.

**Additional file 2: Figure S1a-l.** The primary adherent monolayer cells derived from bone marrow displayed a spindle-shape similar to fibroblasts after culture (a-e); Flow cytometric analysis of MSCs derived from mouse bone marrow at passage three (F-I); Body weight change and liver/body ratio of mice (j-l). Bars =100 μm, seven mice per group were used. ^*^P < 0.05, ^**^P < 0.01, ^***^P < 0.001.

**Additional file 3: Figure S2 a-d.** Adipogenic (a,b) and osteogenic (c,d) differentiations of bone marrow-derived MSCs before and after staining. Bars = 100 μm.

**Additional file 4: Figure S3 a-f.** Normal (a) and transfected BM-MSCs by scrambled siRNAs (b) or TSG-6 siRNAs (c) as confirmed by RT-qPCR (d-f). Bars =100 μm.

**Additional file 5: Figure S4a-l.** Flow cytometric analysis of hepatic infiltration by neutrophils with anti-Ly6G antibodies conjugated with fluorescein isothiocyanate (FITC). LyG6+ cells from the control (a), AH (b,g), AH+MSCs (c,h), AH+MSCs+AG490 (d), AH+sc-MSCs (i), AH+siTSG-6-MSCs (j), and AH+rmTSG-6 (k) groups and analytic results (e,l); MPO activity(f). Five mice per group were used. ^*^P < 0.05, ^**^P < 0.01, ^***^P < 0.001.

**Additional file 6: Figure S5a-k.** Flow cytometric analysis of hepatic infiltration by monocytes with anti-CD11b antibodies conjugated with allophycocyanin (APC). CD11b+ cells from the control (a), AH (b,f), AH+MSCs (c,g), AH+MSCs+AG490 (d), AH+sc-MSCs (h), AH+siTSG-6-MSCs (i), and AH+rmTSG-6 (j) groups and analytic results (e,k). Five mice per group were used. ^*^P < 0.05, ^**^P < 0.01, ^***^P < 0.001.

**Additional file 7: Figure S6a-c.** Low frequency of SYR + cells were detected in the inflammed liver of mice transplanted with MSCs (a) or transinfected MSCs (b,c). Seven mice per group were used. Bars =20 μm.

**Additional file 8: Figure S7a-h.** Intraperitoneal infusion of AG490 plus MSCs led to more pronounced improvement of liver injury than MSCs alone. ALT and AST levels (a,b), serum TC and TG (c,d), hepatic TC and TG (e,f), hepatic MDA and GSH (g,h). Seven mice per group were used. ^*^P < 0.05, ^**^P < 0.01, ^***^P < 0.001.

**Additional file 9: Tables S1, S2.** Primer sequences for quantitative real-time PCR.

## Data Availability

The datasets used and/or analyzed during the current study are available from the corresponding author on reasonable request.

## Abbreviations

AH: Alcoholic hepatitis
ALD: Alcoholic liver disease
ALT: Alanine aminotransferase
AST: Aspartate aminotransferase
BM: Bone marrow
Cox: Cyclo-oxygenase
ELISA: Enzyme-linked immunosorbent assay
GFP: Green fluorescent protein
GSH: Glutathione
IL: Interleukin
MAPKs: Mitogen-activated protein kinase
MDA: Malondialdehyde
MPO: Myeloperoxidase
MSCs: Mesenchymal stem cells
NF-κB: Nuclear factor (NF)-κB
NIAAA: The National Institute on Alcohol Abuse and Alcoholism
RT-qPCR: Reverse transcription quantitative PCR
sc-MSCs: Scrambled-siRNA-MSCs
SD: Standard deviation
siRNA: Small interfering RNA
STAT3: Signal transducer and activator of transcription 3
TC: Total cholesterol
TG: Total triglyceride
TNF: Tumor necrosis factor
TSG-6: TNF-α-stimulated gene/protein 6
