# Supplementary material for: Mesenchymal stem cells alleviate liver injury induced by chronic-binge ethanol feeding in mice via release of TSG6 and suppression of STAT3 activation
Source: Stem Cell Res Ther. 2020 Jan 13;11:24. doi: 10.1186/s13287-019-1547-8 (PMC6958598; doi:10.1186/s13287-019-1547-8)
Supplement: Supplementary file 1 — Additional file 1:. Supplementary materials. Mouse bone marrow-derived mesenchymal stem cells: isolation, culture, characterization and transfection; sample collection, storage and various assay. [file 13287_2019_1547_MOESM1_ESM.pdf]

Supplemental materials

Supplemental methods

Isolation and culture of mouse bone marrow mesenchymal stem cells (BM-MSCs).

BM-MSCs were isolated by flushing femurs and tibias of mice. The flushed media was collected and centrifuged at 1000 rpm for 3 minutes. The cell pellet was resuspended and plated in 25-cm^2 culture flasks with Dulbecco's modified Eagle medium:Nutrient Mixture F-12 (DMEM/F12; Gibco, Grand Island, NY, USA) supplemented with 10% heat-inactivated fetal bovine serum (FBS) (Gibco, Grand Island, NY, USA), 100 U/ml penicillin, 100 μg/ml streptomycin (Hyclone, Logan, UT, USA), and 10 nM GlutaMAX-I supplement (Invitrogen, Raritan, NJ, USA), and were maintained in a humidified 5%CO2 atmosphere at 37℃. After a 24-hour incubation, the non-adherent cells were removed and fresh culture medium was added to the flasks. The culture medium was changed twice a week. After 70%-90% confluence was achieved, the adherent cells were trypsinized with 0.25% trypsin/EDTA (Gibco, Grand Island, NY, USA) and subcultured for further expansion.

Phenotypic characterization by flow cytometry.

Cells at passage 2-3 were harvested and resuspended at a concentration of 1×10^6 cells/100μl after washing with phosphate-buffered saline (PBS; Sigma, St. Louis, MO, USA)-containing 0.5% (w/v) bovine serum albumin (BSA; Sigma-Aldrich, St. Louis, MO, USA). They were incubated at room temperature for 30 minutes in the dark with

rat anti-mouse antibodies against CD90-phycoerythrin (PE) (Abcam, Cambridge, MA, USA), CD29-fluorescein isothiocyanate (FITC) (Abcam, Cambridge, MA, USA), CD45-TITC (Invitrogen, Carlsbad, CA, USA) and CD105-TITC (Invitrogen, Carlsbad, CA, USA). After washing twice with PBS, labeled cells were assayed using a Partec GmbH CyFlow Space system (Partec, Augsburg, Germany).

Differentiation studies.

Resuspended cells derived from mouse bone marrow (passages 2 to 3) were grown at a density of 2 × 10^4/well on six-well plates (Corning, NY, USA) and cultured in special conditional adipogenic medium (Cyagen Biosciences, Guanzhou, China) according to the manufacturer's instruction. After 3 weeks, adipogenesis was assessed by oil red O staining (Sinopharm Chemical Reagent Co., Shanghai, Ltd, China). Similarly, osteogenic differentiation was performed by culturing cells (passages 2 to 3) in special conditional osteogenic medium (Cyagen Biosciences, Guanzhou, China) according to the manufacturer's protocol. Three weeks later, osteogenic differentiation was confirmed by Alizarin Red S staining (Sinopharm Chemical Reagent Co., Shanghai, Ltd, China). Cells cultured in normal growth medium were used as controls.

Transfection of BM-MSCs with TSG-6 siRNA.

BM-MSCs were transfected as previously described [1,2]. Briefly, 2 × 10^5 MSCs were plated in 6-well plates (Corning, NY, USA) or T-75 culture flask (Corning, NY,

USA) and cultured for 24 hours. The cells were then transfected with TSG-6 small interfering RNAs (siRNAs) or scrambled siRNA using the lentiviral expression vector (Genomeditech, Shanghai, China) carrying green fluorescent protein (GFP) according to the manufacturer's instructions. 12 hours later, half of the culture medium was replaced, and the cells were allowed to grow for 3-4 days. Successful transfection with Lenti-scramble-siRNA and Lenti-TSG-6-siRNA was confirmed by observing the GFP signals using the inverted fluorescence microscope (OLYMPUS CX41, Tokyo, Japan). To assess the efficiency of the TSG-6 knockdown, RNA was extracted from aliquots of harvested cells and analyzed for TSG-6 expression using real-time quantitative-PCR (RT-qPCR). Then MSCs in T-75 culture bottles were harvested with 0.25% trypsin, and resuspended at 1×10^7 cells in 1 ml sterile saline for the subsequent injection. The primer sequences used in this confirmatory analysis was presented in Table sS11.

Sample collection and storage.

9h after the last garage, all mice were anesthetized, and blood samples were collected in 1.5ml heparin-free EP tubes after eyeball extractions. Serum was obtained by centrifugation at 1500rpm for 10min at 4℃, and stored at −80℃until further analysis. Peritoneal fluid lavage sample was harvested by injecting 3 ml of PBS into the peritoneal cavity followed by gentle massage of the abdomen and collecting the lavage fluid with a sterile syringe. The lavage sample was also centrifuged at 1500rpm for 10 min at 4 °C, and the supernatant was aliquoted and stored at –80℃.

Meanwhile, the cell pellets were flash frozen in liquid nitrogen, and also stored at –80 ℃ until further processing. Every liver specimen was weighed and subsequently divided into three pieces. Two pieces were flash frozen in liquid nitrogen, and stored at –80℃, and one piece was fixed in 10% paraformaldehyde until further assays.

Biochemical assay.

Serum concentrations of alanine aminotransferase (ALT), aspartate aminotransferases (AST), triglycerides (TG), and total cholesterol (CHOL) were analyzed using spectrophotometric assay kits (NanJing JianCheng Bioengineering Institute, Jiangsu, China) according to the recommended protocols. Hepatic levels of malondialdehyde (MDA) and glutathione (GSH) were determined using MDA spectrophotometric assay kit (NanJing JianCheng Bioengineering Institute, Jiangsu, China) and GSH assay Kit (NanJing JianCheng Bioengineering Institute, Jiangsu, China), according to the manufacturer's instructions. Hepatic lipids were extracted from liver homogenate with a 2:1 chloroform:methanol mixture and measured using the triglyceride assay kit or cholesterol assay kit (NanJing JianCheng Bioengineering Institute, Jiangsu, China) according to the manufacturer's instructions.

Myeloperoxidase (MPO) activity analysis.

After homogenization, hepatic MPO activity were measured with a U-3010 UV-Vis Spectrophotometer (HITACHI, Tokyo, Japan) using MPO assay kit (NanJing JianCheng Bioengineering Institute, Jiangsu, China) according to the manufacturer's

protocol. MPO activity was expressed in units per gram of wet tissues, where one unit represents the enzyme activity required to degrade 1μM H_2O_2/min/ml at 24 °C.

Liver histology, and histology scores for hematoxylin and eosin (H&E) staining and oil red O stain.

Liver specimens were fixed in 10% paraformaldehyde, embedded in paraffin and cut into 5μm sections. Specimens were dewaxed, hydrated, and stained with standard H&E for routine histology. For assessment of lipid accumulation, 10 μm sections were cut from frozen samples and stained with Oil Red O (Sinopharm Chemical Reagent Co., Shanghai, Ltd, China). Histology scores for H&E-stained sections was performed by a pathologist who were blind to the study protocol as previously described [3,4]. Briefly, the hepatic steatosis, hepatocyte ballooning and necroinflammatory activity were scored and averged from 10 random 200× fields per liver section from the same mouse as shown in Table S2. Hepatocyte ballooning was identified when they were enlarged to more than twice the size of their neighboring cells. For assessment of lipid accumulation, 10 μm sections were cut from frozen samples and stained with Oil Red O (Sinopharm Chemical Reagent Co., Shanghai, Ltd, China), and morphometric assessment was performed using Image Pro plus 7.0 Software (Media Cybernetics, Bethesda, MD).

Liver immunofluorescence analysis

After antigen retrieval, the sections were blocked with 2% bovine serum albumin and

0.3% Triton X-100 (Sigma-Aldrich, St. Louis, MO, USA) for 60min. The sections were then incubated overnight at 4℃ in the dark with rat anti-mouse SRY-PE (Santa Cruz Biotechnology, Dallas, TX, USA). The sections were washed three times and mounted in a Vectashield mounting medium containing 4',6-diamidino-2-phenylindole (Vector Laboratories, Burlingame, CA, USA). The samples were observed using a Laser scanning confocal microscope (Leica SP5, Wetzlar, Germany). Immunoreactive cells were calculated in 10 random fields at 100× magnification per mouse.

RNA extraction, cDNA synthesis, and quantitative real-time PCR.

Total RNA was extracted from homogenized BM-MSCs, peritoneal lavage cell pellets or mouse liver tissues using TRIzol™ Reagent (Invitrogen, Carlsbad, CA, USA) according to the manufacturer's instructions. cDNA was synthesized using RevertAidTM First Strand cDNA Synthesis Kit (Thermo Fisher Scientific, Waltham, MA, USA). Real-time quantitative PCR analyses were performed in triplicate for each sample by using SYBR Green master mix (KAPA, Wilmington, MA, USA) in an ABI stepone plus real-time PCR System (Applied Biosystems, San Francisco, CA, USA). β-actin level was employed as an internal control. The standard $2^{-\Delta\Delta Ct}$ method was used to calculate relative expression levels of various genes. Primer sequences used in this study were also listed in Table sS1.

Western Blot Analysis

BM-MSCs, peritoneal lavage cells, and mouse liver tissues were lysed with RIPA lysis buffer (Beyotime, Shanghai, China). Whole extracts were prepared, and protein concentrations were evaluated using a bicinchoninic acid (BCA) protein assay kit (Beyotime, Shanghai, China). Total protein (80μg) from each sample was separated by sodium dodecyl sulfate-polyacrylamide gel electrophoresis (SDS-PAGE) and blotted onto a polyvinylidene difluoride membrane (PVDF) (Millipore, Billerica, MA, USA). After blockade of non-specific protein binding with 5% non-fat dry milk in Tris-Buffered Saline and Tween 20 (TBST), the membrane was incubated for 1 h with primary antibodies at a dilution of 1:1000-2000: rabbit anti-mouse interleukin (IL)-6, tumor necrosis factor (TNF)-α, TNF-α stimulatted gene/protein (TSG)-6, cyclo-oxygenase (Cox)-2, and signal transducer and activator of transcription 3 (STAT3) (Proteintech, Chicago, IL, USA), IL-10 (Beijing Bioss biotechnology co., LTD, China), p-STAT3 (Abcam, Cambridge, MA, USA) and β-actin (Beijing zhongshan jinqiao biotechnology co. LTD, China). Secondary antibodies using horseradish peroxidase (HRP)-conjugated goat anti-mouse antibodies (Cell Signaling Technology, Beverly, MA, USA) were applied at room temperature for 1 hour. After extensive washing in TBST, the immunoblots were processed with distilled water and enhanced chemiluminescent (ECL) kit (Millipore, Billerica, MA, USA). Proteins were visualized by BIO-RAD Gel Doc XR (Bio-Rad, Hercules, CA, USA). All experiments were repeated three times.

Enzyme-linked immunosorbent assay (ELISA)

The cytokine concentrations in the mouse serum and peritoneal lavage supernatants were determined using mouse ELISA Kits: IL-6, IL-10, TNF-α and Prostaglandin E2 (PGE2) (Elabscience Biotechnology Co.,Ltd, Wuhan, China), indoleamine 2,3-dioxygenase 1 (IDO1) (CUSABIO BIOTECH CO.,Ltd, Wuhan, China), and TSG-6 (Shanghai Enzyme-linked Biotechnology Co.,Ltd,China). Assays were performed according to the manufacturer' instructions. Briefly, all reagents, samples and standards were prepared as instructed. Add 100 μl standard or sample to each well and incubated for 90min at 37℃. Then add 100 μl biotinylated detection antibody and incubate for 1 hour at 37°C. Aspirate and wash 3 times. Add 100 μl HRP Conjugate and incubate for 30 min at 37°C. Aspirate and wash 5 times. Add 90 μl of substrate reagent and incubate for 15 min at 37°C. Then add 50 μl stop solution and the absorbance at 450nm of each well was immediately measured by a microplate reader (Molecular Devices SpectraMax 190, Sunnyvale, CA, USA).

Isolation of hepatic leukocytes and flow cytometric analysis.

The liver tissue was cut into small pieces with surgical scissors and placed in Petri dishes containing PBS (Sigma-Aldrich, St. Louis, MO, USA). Small pieces (about $1mm^3$) were ground and transferred to a centrifuge tube for centrifugation at 1000rpm for 5min at 4℃. Depending on the amount of cell pellet, 5-6 times (3-5ml) 0.25% trypsin/EDTA (Gibco, Grand Island, NY, USA) was added and digested at 37℃ for 20min. Shaking and pipetting once every 5min was performed. Digestion was terminated by adding 3-5ml serum-containing culture medium. Large undigested

tissue blocks were removed with a 150 μm cell strainer. The cell suspension was centrifuged at 1000rpm for 5min at 4℃, and the supernatant was discarded. After being resuspended in 5ml DMEM/F12 (Gibco, Grand Island, NY, USA), the cell pellet was centrifuged again at 1000rpm for 5min at 4℃ and the supernatant was discarded. The resultant leukocyte pellet was resuspended in 100μl PBS, and incubated with 5μl designated antibodies in dark at room temperature for 30min. Flow cytometric analysis of each sample was performed using a Partec GmbH CyFlow Space system (Partec, Augsburg, Germany). The following rat antibodies against mouse were used: anti-Ly-6G-FITC (Invitrogen, Raritan, NJ, USA), anti-CD11b-allophycocyanin (APC) (Abcam, Cambridge, MA, USA), and anti-CD206-PE (Invitrogen, Raritan, NJ, USA).

Supplemental results

Morphology, phenotype and differentiation profiles of BM-MSCs.

The primary adherent monolayer cells derived from bone marrow of male mice formed many colonies after 6 to 10 days. They displayed a fibroblastic morphology (supplemental fig.1a-e), and were passed every 3 to 4 days. The immunophenotype of culture-expanded cells (passages 2 to 3) were assayed by flow cytometry, which revealed that they were positive for CD29 (82.84%), CD105 (85.27%) and CD90 (84.17%), but negative for CD45 (0.65%) (supplemental fig.1f-i). After culture in adipogenic medium for 15 days, subcultured cells derived from male mouse bone marrows formed large number of lipid droplets and vacuoles in the cytoplasm, and

were stained positive by oil red O staining (supplemental fig.2a,b). After culture in osteogenic medium for 21 days, subcultured cells exhibited plenty of calcified bone nodules, and were stained positive by Alizarin Red S staining (supplemental fig.2c,d). These features were consistent with the typical BM-MSCs, but not hematopoietic cells [2,5-57].

TSG-6 expression was knocked down after transfection.

After transfection with small interfering RNAs (siRNAs), the MSCs transfected with TSG-6 (siTSG-6-MSCs) or scrambled siRNAs (sc-MSCs) maintained their fibroblast-like shape and proliferative ability in vitro (Supplemental Fig.3a-c). And TSG-6 expression in bone marrow-derived MSCs was markedly knocked down (supplemental fig.3d-f) compared to normal MSCs (by 75.4%) or sc-MSCs (by 74.2%).

References

1. Liu L, Song H, Duan H, Chai J, Yang J, Li X, et al. TSG-6 secreted by human umbilical cord-MSCs attenuates severe burn-induced excessive inflammation via inhibiting activations of P38 and JNK signaling. Sci Rep. 2016;6:30121.

2. Liu Y, Zhang R, Yan K, Chen F, Huang W, Lv B, et al. Mesenchymal stem cells inhibit lipopolysaccharide-induced inflammatory responses of BV2 microglial cells through TSG-6.J Neuroinflammation. 2014;11:135.

3. Ge X, Leung TM, Arriazu E, Lu Y, Urtasun R, Christensen B, et al. Osteopontin binding to lipopolysaccharide lowers tumor necrosis factor-α and prevents early alcohol-induced liver injury in mice.Hepatology. 2014;59(4):1600-16.

4. Hubscher SG. Histological assessment of non-alcoholic fatty liver disease.Histopathology.2006;49(5):450-65.

5. Dominici M, Le Blanc K, Mueller I, Slaper-Cortenbach I, Marini F, Krause D, et al. Minimal criteria for defining multipotent mesenchymal stromal cells. The International Society for Cellular Therapy position statement. Cytotherapy. 2006; 8(4): 315-7.

6. Sun T, Gao GZ, Li RF, Li X, Li DW, Wu SS, et al. Bone marrow-derived mesenchymal stem cell transplantation ameliorates oxidative stress and restores intestinal mucosal permeability in chemically induced colitis in mice. Am J Transl Res. 2015;7(5):891-901. eCollection 2015.

7. Sala E, Genua M, Petti L, Anselmo A, Arena V, Cibella J, et al. Mesenchymal Stem Cells Reduce Colitis in Mice via Release of TSG6, Independently of Their Localization to the Intestine. Gastroenterology. 2015;149(1):163-76.e20.
