# Supplementary material for: Mesenchymal stem cells alleviate liver injury induced by chronic-binge ethanol feeding in mice via release of TSG6 and suppression of STAT3 activation
Source: Stem Cell Res Ther. 2020 Jan 13;11:24. doi: 10.1186/s13287-019-1547-8 (PMC6958598; doi:10.1186/s13287-019-1547-8)
Supplement: Supplementary file 9 — Additional file 9: Tables S1, S2. Primer sequences for quantitative real-time PCR. [file 13287_2019_1547_MOESM9_ESM.pdf]

Table S1. Primer sequences for quantitative real-time PCR.

Target genes	Forward primer (5'…3')	Reverse primer (5'…3')	Length （bp）
TSG-6	CTTGGCTGACTATGTAGA	TTCCTGTGCTAATGATGT	106
IL-6	ACCTGTCTATACCACTTC	GCATCATCGTTGTTCATA	117
IL-10	AGCAGGTGAAGAGTGATT	GCAGTTGATGAAGATGTCA	83
TNF-α	TTCTGTCTACTGAACTTC	CCATAGAACTGATGAGAG	82
COX2	GTCTGGAACATTGTGAAC	GTAGTAGGAGAGGTTGGA	153
β-actin	TATGGAATCCTGTGGCATC	GTGTTGGCATAGAGGTCTT	87

Table S2 Histological scoring system.

Pathological features	Severity or extent in a 100x field	Scores (0-3)
Hepatic steatosis	<5% hepatocytes involved	0
	5–33% hepatocytes involved	1
	33–66% hepatocytes involved	2
	>66% hepatocytes involved	3
Hepatocyte ballooning	None	0
	Few ballooned cells	1
	Many cells/prominent ballooning	2
Necroinflammatory activity	None	0
	<2 foci per 100× field	1
	2-4 foci per 100x field	2

	5-10 foci per 100× field	3
	>10 foci per 100× field	4
